# Novel Applications of Laser Doppler Vibration Measurements to Medical Imaging

**DOI:** 10.1007/s11220-013-0077-1

**Published:** 2013-08-13

**Authors:** Habib Tabatabai, David E. Oliver, John W. Rohrbaugh, Christopher Papadopoulos

**Affiliations:** 1 Department of Civil Engineering and Mechanics, University of Wisconsin-Milwaukee, 3200 N Cramer Street, Milwaukee, WI 53211 USA; 2 Polytec, Inc., 25 South Street, Suite A, Hopkinton, MA 01748 USA; 3 Washington University School of Medicine, Campus Box 8134, One Brookings Drive, St. Louis, MO 63130 USA; 4 Department of Engineering Science and Materials, University of Puerto Rico, Mayagüez, Call Box 9000, Mayagüez, PR 00681-9000 USA

**Keywords:** Laser Doppler Vibrometry, Tissue vibration measurement, Blood flow imaging

## Abstract

Laser Doppler Vibrometry (LDV) has been widely used in engineering applications involving non-contact vibration and sound measurements. This technique has also been used in some biomedical applications including hearing research. The detectable frequencies are in the range of near-DC to 1 GHz or higher. This paper reviews applications of LDV in biomedical engineering and proposes new medical imaging applications based on measuring surface vibrations of tissues and organs. Tests were conducted on human skin using single point and scanning laser vibrometers. These tests suggest that skin vibrations due to the forcing excitation from the heart can be used in imaging of blood flow. The results of these tests illustrate the potential of such vibration measurements in a variety of diagnostic medical imaging applications including blood flow/restrictions, real-time monitoring of blood pressure variations, wound healing, muscle movements, etc. The fact that the measurements can be conducted remotely (non-contact) is an important benefit that adds to the promise of this approach.

**Electronic supplementary material:**

The online version of this article (doi:10.1007/s11220-013-0077-1) contains supplementary material, which is available to authorized users.

## Introduction

This paper reviews applications of Laser Doppler Vibrometry (LDV) in biomedical engineering and proposes new medical imaging applications based on measuring surface vibrations of tissues and organs. LDV is a long-standing technique for non-contact measurement of surface vibrations in a number of fields including mechanical, civil, automotive, aerospace, manufacturing, and biomedical engineering. The vibration measurement “sensor” in LDV is not coupled (attached) to the measurement surface. Rather, a laser beam is aimed at the target location on a surface and the displacement and/or velocity of the surface along the direction of the laser beam is measured through the Doppler effect and the principles of interferometry. The detectable frequencies are in the range of near-DC to 1 GHz or higher.

The Doppler effect is the apparent frequency change (shift) noticed by an observer when the source of a wave is moving relative to the observer. This frequency shift is proportional to the velocity of relative movement between the observer and the wave source. This effect can be combined with the principles of interferometry to accurately measure surface velocity (using velocity decoding) down to 10 nm/s/√Hz or displacements (using displacement decoding) down to sub-picometer resolution per √Hz [1, 2].

In a classical interferometer, when two coherent light beams (lasers) combine, the resulting intensity has a component (an “interference” term) that is related to the difference in path lengths of the two beams. One of the two laser beams is called the reference and the other the measurement beam. An interferometer is used to detect dark and bright (fringe) patterns corresponding to the number of half wavelengths (of the laser beam) that make up the difference in path length between the reference and measurement beams.

**Fig. 1:**
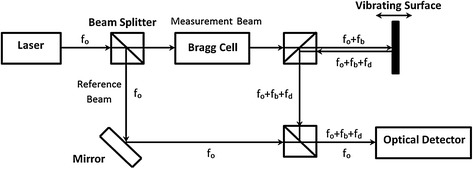
Example of Laser Doppler Vibrometry

Measurements can be made from large distances (up to tens or hundreds of feet). The laser beam is typically a low-power laser (Class 2 helium-neon visible) with a wavelength of 633 nm. The Class 2 laser used in this approach is considered safe. Although the blink response of the human eye is typically considered sufficient to avoid eye damage, eyes may still need to be protected during scans to prevent accidental exposure. Figure 1 shows an example of the basic concept used in this approach.

A laser Doppler vibrometer is a special kind of optical interferometer [2]. Within the LDV hardware, the laser beam is first split into two beams (reference and measurement). The measurement beam with frequency *f*_*o*_ is focused onto the measurement point on the test surface. The reflected beam, experiencing a frequency shift *f*_*d*_, proportional to the velocity of the test surface, is re-combined with the reference beam at an optical detector. A Bragg Cell is used to shift the frequency of the reference laser by *f*_*b*_ (typically 40 MHz). Without a Bragg Cell, the system would not be able to distinguish whether the detected movement is towards or away from the laser. This 40 MHz signal ±*f*_*d*_ is demodulated electronically to provide a voltage output proportional to instantaneous velocity. A voltage proportional to displacement can also be provided by decoding the relative phase between the carrier signal driving the Bragg cell and the photo-detector signal. Commercially available laser vibrometers can achieve a velocity resolution down to 10 nm/s/√Hz and a displacement resolution of less than 1 pm/√Hz, depending on the decoding electronics and to some extent on the amount of light being backscattered from the test surface and any relative motion of the laser. Depending on the optoelectronic configuration, velocities in excess of 30 m/s can be measured and the operating vibration frequencies span from near-DC to >1 GHz.

Commercial laser vibrometers can be single point (LDV), scanning (SLDV), or 3-dimensional scanning (3D-SLDV) systems. Single point vibrometers measure the velocity of a point on a surface along the direction of the laser. Scanning systems can measure vibrations on a grid of points on a surface, and can generate modal vibration information. The 3D-SLDV system consists of three SLDV systems that can simultaneously scan over a planar or non-planar (curved) test surface and provide complete 3-D vibration and modal information [3].

## Engineering Applications of LDV

Before discussing the use of LDV in imaging, we first provide a brief summary of the established use of LDV in engineering applications. Laser vibrometers are widely used in automotive, aerospace, medical, biological, ultrasonic, microstructural, and manufacturing applications, largely for vibration measurements, system identification, testing, and damage detection. LDV provides a non-contact alternative in cases in which attachment of sensors is not practical or can influence the structure response characteristics, such as in lightweight structures, high-temperature applications, or liquid surfaces.

**Fig. 2:**
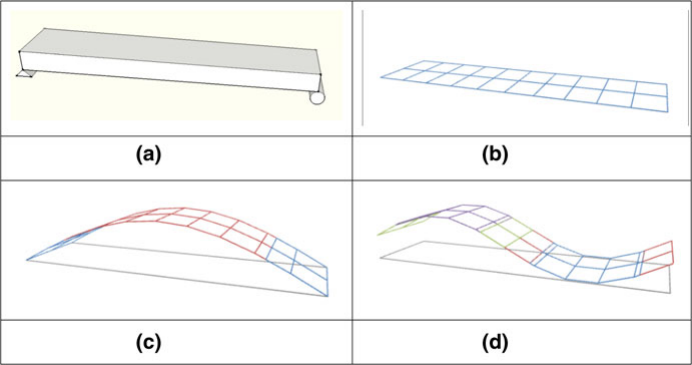
Example of a structure (**a**), its test grid (**b**), its first mode (**c**) and second mode (**d**) vibration shapes

One application of LDV is in “experimental modal analysis” in which measurements of natural frequencies, associated characteristic vibration shapes (mode shapes), and damping levels of a structure are determined. A general way to accomplish this is to specify or import a set of measurement/excitation points (grid points) on the surface of a structure. An excitation (typically a dynamic force or impulse) is then applied, and the responses (e.g., displacement, velocity, or acceleration) are measured sequentially at every grid point. The ratios of the response at each grid point to the excitation at the same or other grid point are calculated in the frequency domain. These ratios (functions) are called Frequency Response Functions (FRF). FRFs are used to generate characteristic vibration shapes (mode shapes) at various natural frequencies. Figure 2 illustrates how grid points can be assigned to a simple beam and the beam’s first two characteristic mode shapes.

In a variation to the basic modal analysis test procedures, ambient vibrations can be used instead of an applied force or impulse. In such cases, the response of a stationary sensor (typically an accelerometer or single point LDV) placed at a grid point can be used in the denominator of the FRF ratio (in place of a measured force excitation). Therefore, a combination of a LDV and SLDV can be used for a completely non-contact modal testing.

Determination of vibration mode shapes is important in diagnostic engineering tests because mode shapes can indicate changes in the structure’s primary mechanical parameters that influence dynamic response (stiffness, mass, and damping) as well as its boundary conditions. A damaged structure (e.g., reduced stiffness) could have a different modal shape and frequency. A modified structure (increased or reduced mass or stiffness) would also result in changes in vibration parameters, which can be detected.

This methodology can be applied to a wide variety of engineering contexts. In automotive applications, assessments of vehicle vibrations over small or large surfaces are made using non-contact SLDV. LDV and SLDV have also been used to assess and mitigate vehicle sounds such as squealing brakes, engine noise, rattles etc. [4–6].

Buligan et al. [7] used LDV to measure movements of hermetic compressor valves during operation. Tabatabai et al. [8] applied LDV for condition assessments of bridge stay cables in a project sponsored by the Federal Highway Administration. This study showed that ambient vibrations of such cables can be measured rapidly and remotely (from distances of up to 200 ft), and vibration frequencies can be determined and subsequently used to estimate cable forces for health monitoring assessments.

Several researchers have used LDV and SLDV for damage assessments in historical and cultural heritage structures, artworks, and Italian frescoes [9–14]. Stark, Erfurt and Tatarin used LDV to measure ultrasonic pulse velocity in early age concrete [15]. Ebert [16] utilized SLDV to measure frequencies and mode shapes associated with solar panels, and Reimers and Wiegers [17] used LDV and SLDV to measure vibration parameters on rotor blades and hubs of wind turbines.

Qiao et al. [18] used SLDV for damage detection in E-glass/epoxy laminated composite beams by measuring displacement mode shapes. Fortner [19] utilized laser vibrometry to assess potential for buckling in rails, and Jenkins and Tampi [20] used SLDV to measure vibrations of circular membranes.

Other applications of LDV include surveillance/remote listening by measuring minute vibrations of surfaces of objects near a source of sound (sensitivity on the order of 1 μm/s) [21, 22]; characterization of the performance of pulse-echo phased ultrasonic transducer arrays used in ultrasound imaging; and study of uniformity and crosstalk between transducer elements [23].

## Biomedical and Bioengineering Applications of LDV

Factors that affect the inherent vibration characteristics (e.g., frequencies and mode shapes) of any “structure” include its mass, stiffness and damping. The vibration response of any such structure is a function of those three parameters as well as any “excitation” (e.g., applied force) that may be applied on the structure. In particular, the vibration response of human tissues and organs are functions of their mass, stiffness, damping, and excitation (e.g., pumping of human heart). Over the centuries, ancient and modern physicians have listened to the sounds of the heart (directly, or in the last 200 years through stethoscopes). They also felt vibrations of human pulse at the wrist or the neck as important diagnostic tools. All such vibrations and sounds can be detected in a non-contact manner through laser Doppler vibrometry. If any of the basic vibration parameters change due to illness, injury, or emotional state, the vibrational response characteristics would change as well.

The LDV approach has been widely used as a measurement tool in several research applications involving hearing [24–34]. These applications include cochlear implant effects on middle ear function, middle ear ossicles, ossicular reconstruction, and prosthesis, hearing loss and mechanical problems in hearing. Commercial LDV systems (such as Polytec’s HLV-1000 Hearing Laser Vibrometer) coupled to a surgical microscope (such as Zeiss OPMI-1) with a micromanipulator for independent alignment of the laser with the point of interest, have been used in experiments assessing the tympanic membrane function at the umbo in a clinical environment. Sokolowski et al. [35] used LDV to measure round window movability, and to assess ossicular chain functioning after malleus stapes assembly reconstruction. Todt et al. [36] studied safety of MRI scanning of implantable hearing devices (floating mass transducers) using LDV.

Felver et al. [37] used LDV to assess vibratory motions of ultrasonic scalers used in dentistry. Lea et al. [38–40] used laser vibrometry for assessments of powered toothbrush vibrations as well as endosonic files used in dentistry. Castellini et al. [41, 42] used laser vibrometry to measure “mobility” of teeth (maximum displacement in response to maximum load).

DeMelis et al. [43–45] have explored cardiac rate and variability by non-contact measurement of the velocity of the skin surface of the chest wall and the neck (optical Vibrocardiography, VCG). The authors performed VCG along with phonocardiography and electrocardiogram (ECG) on ten healthy subjects. They identified “some events of cardiac mechanics, correlating the heart sounds relative to the closure of the mitral valve, and the following closure of the aortic and pulmonary valve with characteristic deflections identifiable on VCG traces.”

Agarwal et al. [46] and Moses et al. [47] at the Mayo Clinic measured changes in aneurysm sac pressure in an in vitro aneurysm model using LDV. Explanted porcine abdominal aortas and nitrile rubber tubes were used to model an aneurysm sac. They concluded that “Noninvasive measurement of changes in aortic aneurysm sac tension is feasible in an in vitro setting using the concept of vibrometry. This could potentially be used to noninvasively detect wall stress in native aneurysms and endotension after endovascular aneurysm repair and to predict the risk of rupture.” Zhang et al. [48], also from the Mayo Clinic, studied coupled vibration of arterial tubes in fluids.

Rixen et al. [49] report on their use of LDV for in vivo measurements of thorax and abdomen surfaces. Yazicioglu et al. [50] studied the vibration of a thin-walled cylindrical viscoelastic tube with internal turbulent flow resulting from a flow constriction. LDV was used to measure the vibration of the tube and the surrounding viscoelastic material [51]. Dahl et al. [52] applied the Laser Doppler vibrometry technique to assess the osseointegration of total ankle prosthesis, and concluded that LDV can “determine the integration of implants non-invasively in the clinical and surgical setting”. LDV has also been used for assessing the performance and kinematics of artificial heart valves [53–55]. Rodini et al. [53] provided external excitation to a valve through the use of a shaker. Vibration measurements were used as boundary conditions for the finite element models, and stress maps were generated [53].

**Fig. 3:**
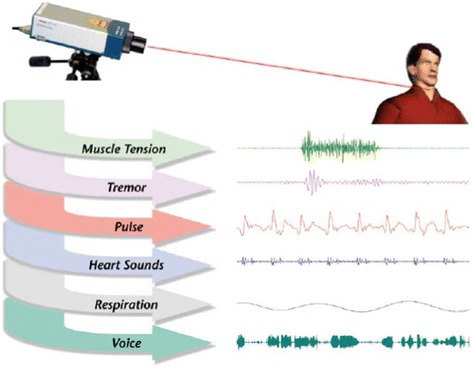
Types of physiological signals that can be recorded

**Fig. 4:**
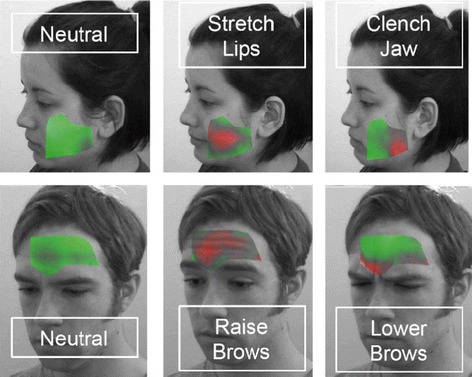
Facial patterns of muscle activity

Multiple investigators at the Washington University [56–59] have studied remote biometric measurements, and developed an LDV system with targeting, tracking and focusing controlled by computer vision methods for assessment of various human physiological parameters including cardiovascular activity (Fig. 3). SLDV is also used to image facial muscle activity related to emotional state (Fig. 4).

**Fig. 5:**
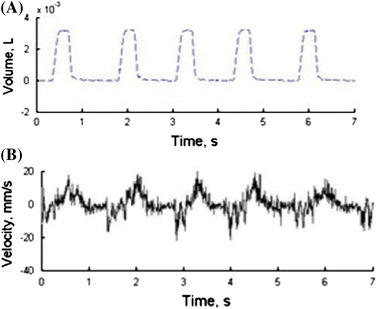
LDV signal measured on an infant’s abdominal wall: volume inspired/expired (**a**) and measured velocity (**b**) [61]

DeMelis et al. [60] used LDV to measure the time from carotid to femoral pulse wave propagation or pulse transit time to estimate the carotid-femoral wave velocity. Scalise et al. [61] used LDV to monitor respiration rates in preterm infants by monitoring abdominal wall movements from outside the incubator. An example of the acquired data is shown in Fig. 5. Schuurman et al. [62] measured vibrations of the abdominal wall to detect aortic aneurysms.

## Proposed Biomedical Imaging Applications of LDV and SLDV

This paper describes early work done by the authors over several years beginning in 2002, and proposes new medical imaging approaches using Laser Doppler Vibrometry. Tests were conducted on human skin using single point and scanning laser vibrometers for biomedical imaging applications. The results of these tests illustrate the high precision and sensitivity of Laser Doppler Vibrometry for vibration measurements, and their significant potential in diagnostic medical imaging applications.

**Fig. 6:**
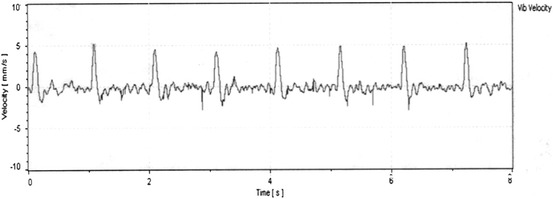
Non-contact single-point laser measurements of vibrations on human wrist due to pump action of the heart

Figure 6 shows unfiltered non-contact single-point LDV velocity measurements on a human wrist (pulse area) from a distance of about 18 inches lasting about 8 s. The entire arm and the wrist were held against a wall. No corrections were made for noise or movements of the wrist. Aside from any involuntary movements of the wrist, the only excitation in this case was the pumping action of the heart, which in turn vibrated the issue in the wrist area. The record shows the velocity (mm/s) of movement of the target point on the skin surface in the direction of the laser beam as a function of time.

This test clearly shows that some basic physiological information can be determined using non-contact LDV. The relatively strong signal shows the potential sensitivity and resolution of such measurements. From a superficial view, Fig. 6 looks similar to an ECG chart. It has similar peaks and valleys. However, the two tests measure different parameters. ECG charts are records of the electrical activity of the heart as measured by electrode pairs (leads) attached to the skin. An ECG measures small electrical change on the skin surface due to depolarization of the heart muscle, and thus does not directly measure actual mechanical effects of the pumping of the heart. On the other hand, the LDV trace in Fig. 6 is a direct result of the vibration induced due to the pumping action of the heart.

From an engineering standpoint, the amplitudes of vibration of the skin and tissue surrounding blood vessels are directly related to the forcing function (i.e. blood pressure). Therefore, the vibration peaks in each cycle can indicate real-time variations in peak blood pressures. Important information can potentially be extracted in real time from this non-contact ability to measure relative changes in blood pressure. For example, simultaneous measurements of vibrations on the left and right wrists can provide information on differences in pressures and arrival times of waves between the left and right sides. Problems such as abnormalities in the aorta can lead to such differences. Simultaneous non-contact measurements could address potential problems associated with sequential conventional pressure measurements.

**Fig. 7:**
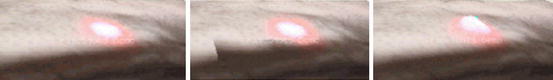
Non-contact scanning laser measurements of vibrations on human wrist showing blood flow under the skin

Figure 7 shows results of a scanning test (SDLV) in which the vibrations (velocity) of skin in the wrist area (near the pulse) was measured as blood pressure changed in the underlying radial artery. A commercial SLDV system (PSV-400-H4 system by Polytec) was used for the scanned measurements. In addition to the SLDV system, a single-point LDV system (OFV-505 optical head from Polytec coupled to OFV-5000 controller electronics) was also used to provide reference measurements for determination of FRFs. The single point laser was stationary and focused on the pulse, while the SLDV beam moved between grid points. The bright spot in Fig. 7 corresponds to where the single point laser illuminated the target point (pulse). Using the equipment software, a rectangular scan area was selected, and grid point spacing was specified. The system includes an embedded video camera that allows the user to draw a scan area on an image of the target. The scan time is a function of the vibration frequency of interest and the number of grid points. In this case, a very low frequency of vibration (frequency of the heart or approximately 1 Hz) was of interest. The total scan time was approximately 5 min in this test, and the range of measured frequencies was from less than 1 Hz to a few kHz. In other cases (such as when ultrasonic frequencies are of interest only), then scan times can be reduced substantially. As stated earlier, the measured FRF functions can be used to determine vibration shapes associated with specific frequencies. In this case, the frequency of interest was approximately 1 Hz.

A video of the test results can be viewed at https://pantherfile.uwm.edu/ht/public/Imaging/wristscan.avi. The vibration shape of the grid area associated with the 1 Hz frequency is superimposed on the image of the wrist from the video camera. In the video and Fig. 7, the skin can be seen to rise and fall as pressures in the arteries within the scan area fluctuate. Although this type of scan may be theoretically possible using ultrasound methods for higher frequencies, ultrasonic methods would require contact and a coupling medium, which could affect vibration response. At specific frequencies, particular vibration patterns may have specific clinical implications that can be evaluated by the medical research community.

**Fig. 8:**
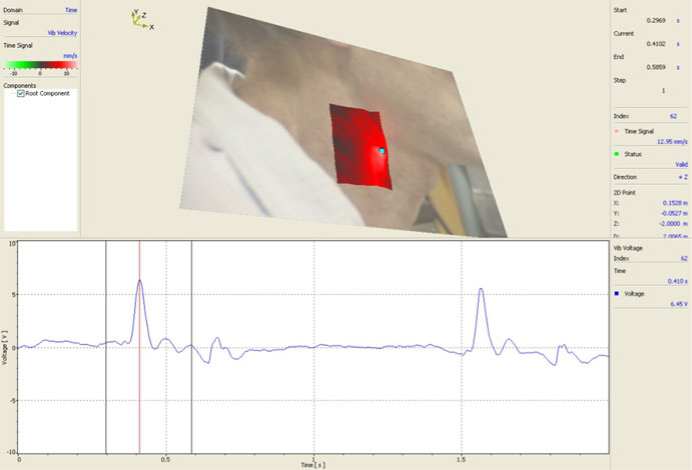
Non-contact scanning and single-point measurements of vibrations on neck area due to pumping action of the heart

**Fig. 9:**
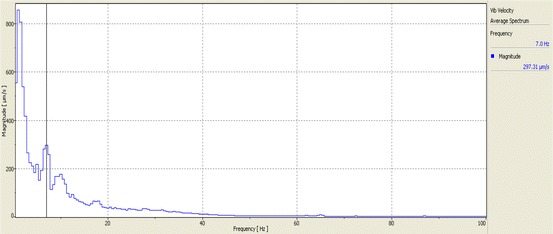
Average velocity spectra (linear scale)

Figure 8 shows results of another scanning test (SLDV) in which the vibrations (velocity) of skin in the neck area (near the pulse) were measured as blood flowed through the carotid artery under the skin. The scanning speed was approximately 2 s per point. A video of this test can also be viewed at https://pantherfile.uwm.edu/ht/public/Imaging/carotid%20time%20animation%20filtered.avi). The time trace in the lower half of Fig. 8 is the data interrogated at one of the scan grid points and includes two pulses. Figure 9 shows frequency spectra for the single point laser response.

The vibration shape in Fig. 8 shows how the skin is moving in response to blood flow. The vibration shape at different frequencies could potentially have important clinical implications. From a structural/vibration engineering standpoint alone, the vibration response of the tissue around the artery (carotid artery in this test) would be different if there were partial blockage within the artery. This is because such restrictions would affect fluid (blood) pressures across the flow restriction. Such blockage would also create higher frequency effects (including turbulent flow effects), which would be reflected in differing surface vibration shapes when observed at those higher frequencies. This new imaging tool can potentially be used by the medical research community to look at the issue of extent of blockage of carotid artery among other effects.

**Fig. 10:**
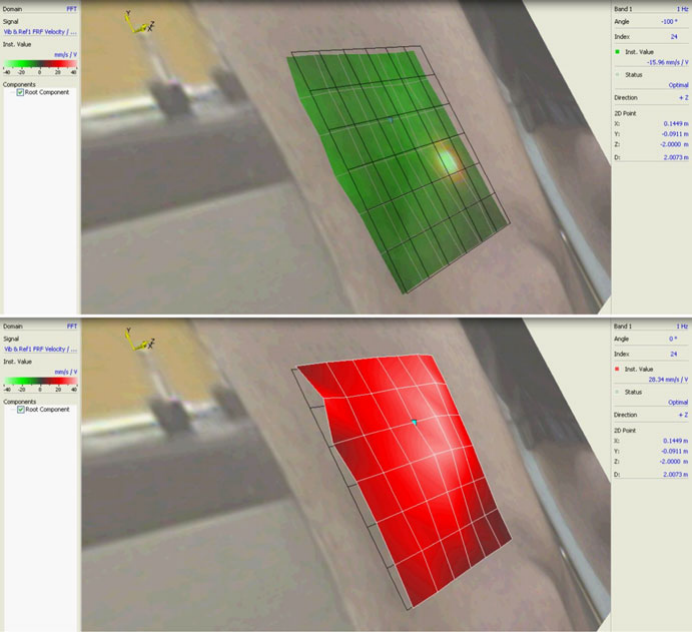
Non-contact scanning laser measurements of vibrations of leg due to muscle flexing above the knee (*Top*: relaxed state, *Bottom*: flexed state)

Other SLDV tests have also been performed by the authors of this paper showing muscle contractions and movements. Figure 10 shows the vibration shape (at 1 Hz) of a grid area above the knee. The test subject was asked to contract and release his leg muscles at about once a second.

Laser Doppler Vibrometry systems can potentially be applied to vibration measurements at a cellular level. There are commercial LDV systems available that are equipped with a microscope (such as Polytec’s MSA-500). Changes affecting mass or stiffness of cells also affect vibration characteristics, which can be monitored. In wound monitoring/healing, restoration of blood flow (flow-induced vibration) and/or regrowth of healthy tissue (stiffness change) could be bases for potential application of LDV or SLDV. Non-contact and real-time measurements of blood pressure variations are another potential area of research using this approach. Further testing and development may make it possible to determine absolute (as well as relative) blood pressures with non-contact LDV. This involves additional testing to develop accurate relationships between vibration and pressure. SLDV monitoring of muscle movements could potentially be used for research on motion injuries. In short, any condition that may affect mass, stiffness or damping of tissues and organs could potentially be monitored using LDV/SLDV.

At the laser wavelength used for the above measurements, 633 nm, a significant amount of incident light penetrates the skin rather than being scattered from the surface. One way to improve the optical return signal, thereby the quality of the measurements, and ensure that the vibration at the surface is being measured rather than interferometric mixing from various depths, would be to employ laser vibrometers operating at 1,550 nm. 1,550 nm light exhibits an absorption coefficient of approximately 1.5 × 10^−3^/cm.

It should be noted that the Laser Doppler Vibrometry approach discussed here is fundamentally different from another Laser Doppler-based approach for imaging of near surface blood flow [63]. In that non-vibrometry approach, blood flow imaging is based on Doppler shifts resulting from moving blood in the microvasculature.

## Summary and Conclusions

In this paper, single point and scanning surface vibration measurements using Laser Doppler Vibrometry are proposed for new medical imaging applications based on principles of vibration testing and structural dynamics. The ability of LDV and SLDV to accurately image vibrations in a wide range of frequencies, from the frequency of the heart or lower up to frequencies in the ultrasound range, is an important factor that can be used to develop a number of medical imaging applications. The fact that the measurements can be conducted remotely (non-contact) is an important benefit that adds to the promise of this approach.

As stated earlier, the forcing excitation from the heart can be used in imaging of blood flow. However, this does not need to be the case in all imaging circumstances. Techniques used in conventional structural vibration testing involving external excitation (such as impulse or shaker inputs of various frequencies or frequency ranges) can also be used. Such excitation can be applied to the body either directly or through the patient’s support structure (chair, arm rest, bed, etc.). When changes in mass, stiffness, or damping of the tissues and organs occur due to a medical condition, the vibration characteristics change as well. The test area can be excited with an excitation source containing the frequencies of interest while the non-contact measurements are taken.

In summary, some of the potential biomedical testing and imaging applications using scanning and/or single point laser Doppler vibrometry include:

- Imaging of blood flow and restrictions (localized or full body). Commercially available two- or three-dimensional scanning LDV systems can be used.
- Non-contact measurement of blood pressure and real-time variations.
- Evaluation of injury or wound healing.
- Measurements of muscle movements and associated vibrations.
- Assessments of repetitive motion injuries.
- Detection of changes in mass, stiffness, or damping of tissue due to various clinical factors.
- Scanning of internal organs (during surgery).

## Electronic supplementary material

Below is the link to the electronic supplementary material.

Supplementary material 1 (AVI 19014 kb)

Supplementary material 2 (AVI 8850 kb)
